# Overexpression of (P)RR in SHR and Renin-Induced HepG2 Cells Leads to Spontaneous Hypertension Combined with Metabolic Dysfunction-Associated Fatty Liver Disease

**DOI:** 10.3390/ijms26136541

**Published:** 2025-07-07

**Authors:** Chen Gao, Xinyi Guo, Lingzhi Zhang, Xueman Lin, Hua Sun

**Affiliations:** State Key Laboratory of Digestive Health, Institute of Materia Medica, Chinese Academy of Medical Sciences and Peking Union Medical College, Beijing 100050, China; gaochen@imm.ac.cn (C.G.); guoxinyirachel@outlook.com (X.G.); zhanglz@imm.ac.cn (L.Z.); linxueman@imm.ac.cn (X.L.)

**Keywords:** (P)RR, (P)RR/ERK/PPARγ, hypertension combined with MAFLD, SHR, renin, lipid accumulation, HRP

## Abstract

Hypertension and metabolic dysfunction-associated fatty liver disease (MAFLD) are both common chronic diseases globally. Nearly half of patients with hypertension are complicated by MAFLD. The mechanisms of the bidirectional promotion between the two remain unclear. The (pro) renin receptor ((P)RR) is one of the classic members of the renin–angiotensin system (RAS) and serves as the receptor for prorenin. Although the role of (P)RR in the induction and progression of hypertension has been extensively studied, its role and underlying mechanisms in MAFLD remain underreported. In this study, we aim to investigate the role of (P)RR in the pathogenesis of hypertension combined with MAFLD. In this study, SHRs were used for the model for hypertension combined with MAFLD. Liver lipid content analysis, liver H&E staining, the detection of (P)RR, ERK and downstream proteins related to fatty acid synthesis and transport, and RNA sequencing and data analysis were performed. In the in vitro experiments, we activated (P)RR using renin and established the lipid deposition model of HepG2 cells induced by renin for the first time. (P)RR was specifically blocked using handle region peptide (HRP), and Nile red fluorescence staining, (P)RR/ERK/PPARγ protein expression analysis, and immunofluorescence were performed to further verify the role of (P)RR in the pathogenesis of hypertension combined with MAFLD. Our results demonstrate that (P)RR plays a role in the development and progression of hypertension combined with MAFLD. The hepatic TG and FFA levels in the SHRs were increased, and the protein expression of the (P)RR/ERK/PPARγ pathway and downstream proteins related to fatty acid synthesis and transport were upregulated. HRP reversed the activation of these proteins and reduced intracellular lipid accumulation. In conclusion, our study first reveals that (P)RR is a potential therapeutic target for hypertension combined with MAFLD. And we found the (P)RR/ERK/PPARγ axis for the first time, which plays an important role in the progression of spontaneous hypertension combined with MAFLD.

## 1. Introduction

Hypertension and MAFLD, as two common chronic diseases, have become significant threats to human health. A substantial body of research [1,2,3] indicates that there is a close and complex intrinsic relationship between hypertension and MAFLD. MAFLD is one of the most concerning chronic liver diseases globally, with a continuously increasing incidence, affecting 38% of the global population [4]. MAFLD encompasses various forms of liver injury, progressing from simple steatosis to more severe and irreversible steatohepatitis with marked inflammatory cell infiltration, and, if left untreated, may continue to deteriorate into fibrosis, cirrhosis, and eventually hepatocellular carcinoma [5,6,7]. The “multiple-hit” hypothesis explains the complex pathogenesis of MAFLD and has gained wide recognition [8]. This hypothesis proposes that obesity, insulin resistance, dietary patterns, environmental factors, and alterations in gut microbiota synergistically drive the initiation and progression of MAFLD. Research indicates that maintaining a balanced diet, controlling energy intake, and implementing personalized nutritional interventions, along with reducing the intake of processed meats, sugar, and alcohol, can help in the prevention and management of MAFLD [9]. MAFLD is closely associated with complications such as hypertension, chronic kidney disease, and type 2 diabetes. Hypertension is one of the key risk factors for cardiovascular disease and has a strong association with metabolic disorders. More than 30% of the global population suffers from hypertension. Numerous research findings [10,11,12] show that the prevalence of MAFLD is significantly higher in hypertensive patients than in normotensive individuals. Nearly 50% of patients with hypertension also have MAFLD [13]. Even in the absence of other influencing factors, early-stage hypertension can promote the onset of MAFLD. Controlling blood pressure or restoring it to normal levels is beneficial in delaying or suppressing MAFLD in non-obese hypertensive patients [14]. Complex pathophysiological mechanisms exist between hypertension and MAFLD, in which inflammation, insulin resistance, and the activation of the RAS play contributory roles. However, the relationship and underlying mechanisms between spontaneous hypertension and MAFLD remain unclear.

In the classical pathways that induce hypertension, the RAS occupies a central role [15]. The (P)RR, as a key component of the RAS, plays an important role in the regulation of the local RAS. (P)RR can activate prorenin, increase renin activity, and promote the production of angiotensin II. (P)RR is a 350-amino acid protein encoded by the ATP6AP2 gene on the X chromosome, and it consists of a transmembrane domain, a cytoplasmic domain, and an extracellular domain [16]. Activation of (P)RR can lead to the excessive activation of the RAS, resulting in vasoconstriction, increased aldosterone secretion, and sympathetic nervous system stimulation, ultimately causing elevated blood pressure. (P)RR can also exert a range of biological effects independent of angiotensin II. Its excessive activation is associated with multi-organ diseases such as heart failure and renal fibrosis [17]. In recent years, (P)RR has been found to be closely associated with MAFLD. Reports have indicated that a specific blockade of (P)RR expression can significantly suppress the synthesis of free fatty acids (FFAs) and regulate intracellular lipid levels in hepatocytes, including triglycerides and cholesterol [18]. However, the precise underlying mechanism remains unclear. In this study, we found that in both the liver of SHRs and the renin-induced HepG2 cells, when (P)RR is activated, its serine and tyrosine residues are phosphorylated, thereby activating the downstream members of the Mitogen Activated Protein Kinase (MAPK) family, ERK1/2 [19]. ERK, a member of the MAPK family, upon activation upregulates peroxisome proliferator-activated receptor gamma (PPARγ), promoting lipid accumulation [20]. The PPAR family is closely associated with glucose and lipid metabolism [21], influencing the expression of sterol regulatory element-binding protein 1c (SREBP-1c) and its downstream genes fatty acid synthase (FAS) and acetyl-CoA carboxylase (ACC) [22]. In this study, our results indicate the presence of the (P)RR/ERK/PPARγ axis in the liver. Mechanistically, activation of (P)RR promotes ERK phosphorylation, which further upregulates PPARγ and SREBP-1c, promoting the expression of ACC, leading to increased synthesis of FFAs and the development of MAFLD. This study reveals the molecular mechanisms underlying spontaneous hypertension combined with MAFLD.

## 2. Results

### 2.1. Systemic and Hepatic RAS Activation in SHRs

**Figure 1:**
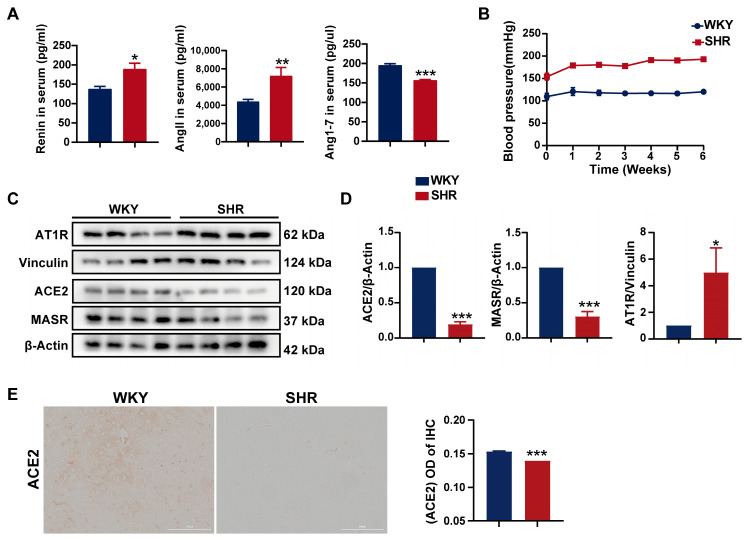
Activation of the systemic and hepatic RAS in SHRs: (**A**) serum levels of renin, AngII, and Ang1–7 in rats; (**B**) systolic blood pressure in rats; (**C**,**D**) protein expression of AT1R, ACE2, and MASR in SHR liver tissue; (**E**) the IHC images and quantification of ACE2 expression (200× magnification). Data are presented as the mean ± SEM, analyzed by an unpaired two-sided *t*-test. * *p* < 0.05, ** *p* < 0.01, *** *p* < 0.001, compared with the WKY group.

SHRs are a commonly used animal model for studying essential hypertension, with RAS activation playing a key role in the pathogenesis of hypertension. Initially, the levels of RAS-related biomarkers were measured in both the WKY and SHR strains. Compared to WKY rats, SHRs exhibited significantly elevated serum levels of renin and angiotensin II (AngII), alongside a notable reduction in angiotensin 1–7 levels (Figure 1A). During the six-week experimental period, SHRs maintained a systolic blood pressure consistently above 150 mmHg (Figure 1B), suggesting that the activation of the systemic RAS contributes to hypertension in SHRs. Furthermore, a significant decrease in ACE2 and MAS receptor protein expression, coupled with a marked increase in AT1 receptor protein expression, was observed in the SHR liver (Figure 1C,D). Immunohistochemistry of SHR liver tissue also revealed a substantial reduction in ACE2 protein expression (Figure 1E). Collectively, these findings indicate a pronounced activation of the hepatic RAS in SHRs.

### 2.2. Significant Liver Injury and Lipid Accumulation in SHRs

**Figure 2:**
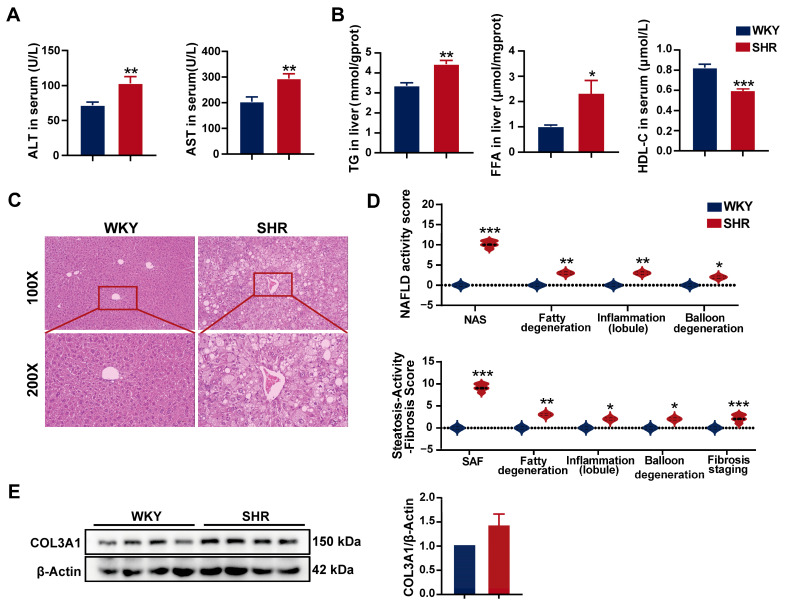
Liver injury and lipid accumulation in SHRs: (**A**) serum liver injury biomarkers in rats; (**B**) hepatic and serum lipid contents in rats; (**C**) representative H&E staining images of SHR liver tissue (*n* = 5). Images with 100× and 200× magnification; (**D**) NAFLD activity score and steatosis activity fibrosis score from H&E staining in the liver; (**E**) protein expression of COL3A1 in SHR liver tissue. Data are presented as the mean ± SEM, analyzed by an unpaired two-sided *t*-test or chi-square test. * *p* < 0.05, ** *p* < 0.01, *** *p* < 0.001, compared with the WKY group.

SHRs are commonly used as a model for hypertension research, with previous reports indicating that SHRs also exhibit hepatic lipid accumulation [23], suggesting the presence of hypertension combined with MAFLD. However, the underlying mechanisms remain poorly understood. In our study, compared to WKY rats, SHRs exhibited significantly elevated serum levels of liver injury biomarkers, including alanine aminotransferase (ALT) and aspartate aminotransferase (AST), indicating notable liver damage (Figure 2A). Furthermore, SHR livers showed a significant increase in triglyceride (TG) and free fatty acid (FFA) contents, while serum high-density lipoprotein cholesterol (HDL-C) levels were significantly reduced (Figure 2B), suggesting substantial hepatic lipid accumulation, consistent with previous findings. Hematoxylin and eosin (H&E) staining revealed prominent cytoplasmic fat vacuoles and edema in the SHR liver (Figure 2C). Both the NAFLD activity score and the steatosis activity fibrosis score of SHR liver tissue were significantly elevated (Figure 2D). Western blot analysis demonstrated no changes in COL3A1 protein expression in SHR liver tissue, suggesting the absence of significant fibrosis (Figure 2E). Taken together, these findings indicate substantial hepatic lipid accumulation and damage in SHRs.

### 2.3. Activation of (P)RR Leads to Lipid Accumulation in Hepatocytes

**Figure 3:**
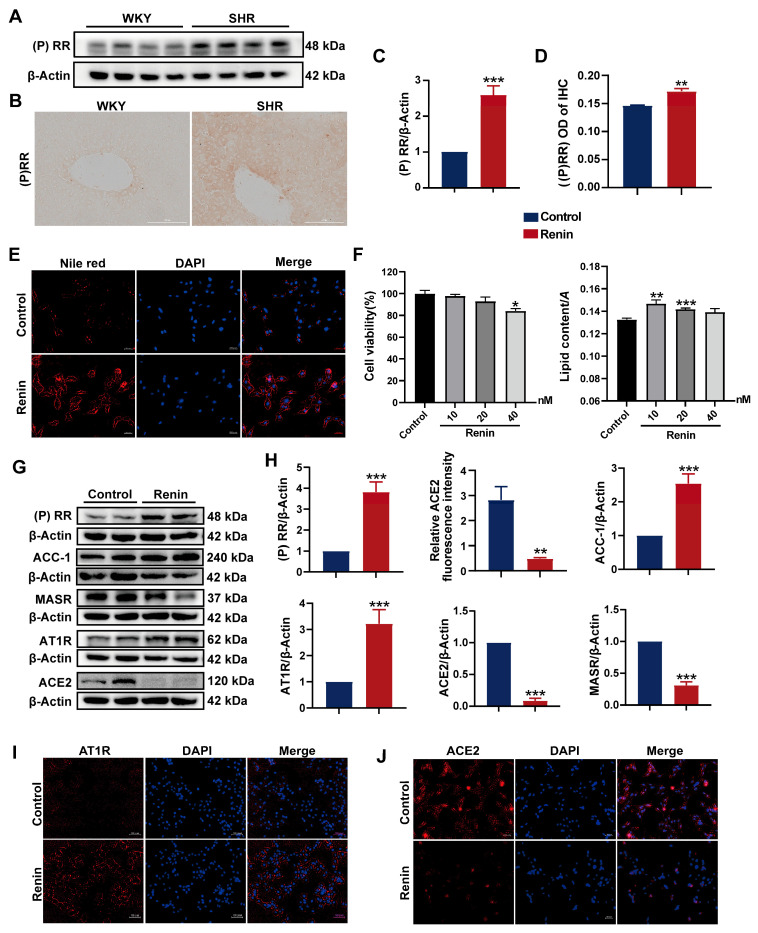
(P)RR activation induces lipid accumulation and RAS activation in HepG2 cells: (**A**,**C**) protein expression of (P)RR in SHR liver tissue; (**B**,**D**) IHC images and quantification of (P)RR expression (200× magnification); (**E**) Nile red (red) staining to observe lipid droplets in HepG2 cells (*n* = 3). DAPI (blue) stains the cell nuclei. Images with 200× magnification; (**F**) cell viability and lipid content in HepG2 cells treated with varying concentrations of renin for 24 h (*n* = 3); (**G**,**H**) protein expression of (P)RR, ACC-1, MASR, AT1R, and ACE2 in HepG2 cells following renin treatment (*n* = 3); (**I**,**J**) relative fluorescence intensity of ACE2 immunofluorescence (red), with DAPI (blue) staining the cell nuclei (*n* = 3). Images with 200× magnification. Data are presented as the mean ± SEM, analyzed by an unpaired two-sided *t*-test. * *p* < 0.05, ** *p* < 0.01, *** *p* < 0.001, compared with the WKY group.

(P)RR, a classical component of the RAS, has been implicated in hepatic lipid accumulation in high-fat diet-fed mice models [24]. Therefore, we investigated the association between (P)RR activation and hepatic lipid accumulation in SHRs. Initially, the protein expression of (P)RR in SHR liver tissue was assessed, revealing a significant upregulation (Figure 3A,C). Immunohistochemical analysis confirmed these findings (Figure 3B,D), indicating marked activation of (P)RR in SHR liver tissue.

Renin can bind to (P)RR and trigger a cascade of extracellular signaling pathways [25]. To further explore the relationship between (P)RR activation and lipid accumulation in hepatocytes, we treated HepG2 cells with renin to activate (P)RR in the cells and examined the changes in lipid content. After 24 h of exposure to 10, 20, and 40 nM renin, no significant changes in cell viability were observed at 10 and 20 nM concentrations. Oil Red O staining and quantitative analysis showed that the lipid droplet content significantly increased in the 10 and 20 nM renin-treated groups compared to the control (Figure 3F). Similar results were obtained using Nile red staining at a 10 nM concentration (Figure 3E). Collectively, the activation of (P)RR increased the lipid content in HepG2 cells.

Activation of (P)RR can further stimulate the RAS by increasing renin activity. We examined the expression of RAS-related proteins in HepG2 cells after renin treatment. Western blot analysis revealed the significant upregulation of (P)RR and AT1R protein expression, while ACE2 and MASR were significantly downregulated. Furthermore, the expression of the fatty acid synthesis-related protein ACC1 was significantly increased (Figure 3G,H). Immunofluorescence analysis showed a significant reduction in ACE2 expression and a marked increase in AT1R expression in HepG2 cells treated with 10 nM renin (Figure 3I,J). These results indicate that the activation of (P)RR activates the RAS pathway in HepG2 cells.

### 2.4. Differential Gene Expression Analysis in SHR Liver

**Figure 4:**
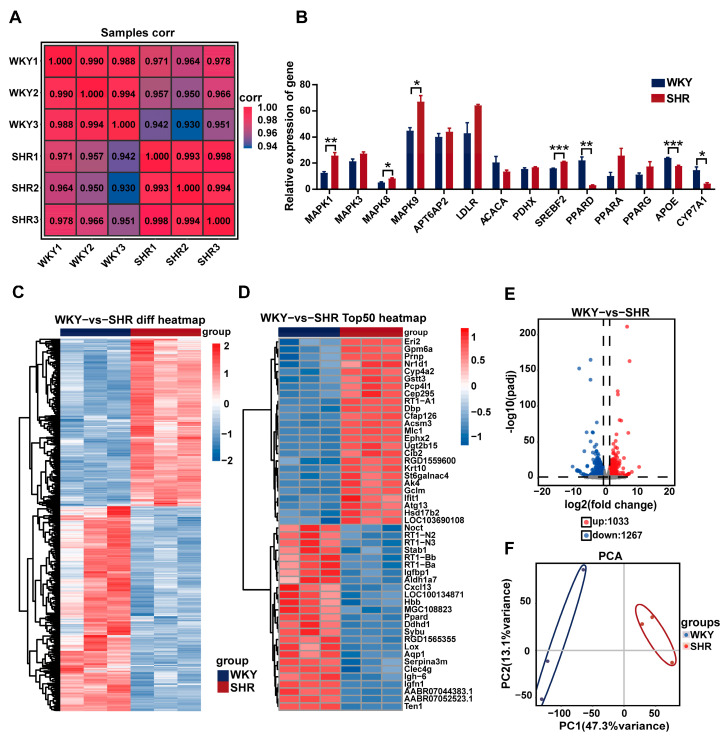
Differential gene expression analysis in SHR liver: (**A**) heatmap of sample correlation; (**B**) quantification of selected differentially expressed genes; (**C**) cluster heatmap of intersecting DEGs between both groups; (**D**) heatmap of the top 50 DEGs between the two groups; (**E**) volcano plot of DEG distribution between both groups; (**F**) PCA of ECSs between both groups. Data are presented as the mean ± SEM, analyzed by an unpaired two-sided *t*-test. * *p* < 0.05, ** *p* < 0.01, *** *p* < 0.001, compared with the WKY group.

RNA-Seq analysis was performed on liver tissue from both the WKY and SHR groups to identify key differential genes. Compared to WKY rats, 2300 protein-coding genes exhibited altered expression in the SHR liver. Among these, 1033 genes were upregulated and 1267 genes were downregulated (Figure 4C–E). Sample correlation and principal component analysis (PCA) revealed significant differences in gene expression between the WKY and SHR liver samples (Figure 4A,F). Notably, genes from the MAPK family, including MAPK1, MAPK8, and MAPK9, were significantly upregulated. The expression of the SREBF2 gene was also significantly increased (Figure 4B). These results suggest a potential link between the MAPK family and hepatic lipid accumulation in SHRs.

### 2.5. Differential Gene Enrich in ERK Cascade and PPAR Pathways in SHR Liver

**Figure 5:**
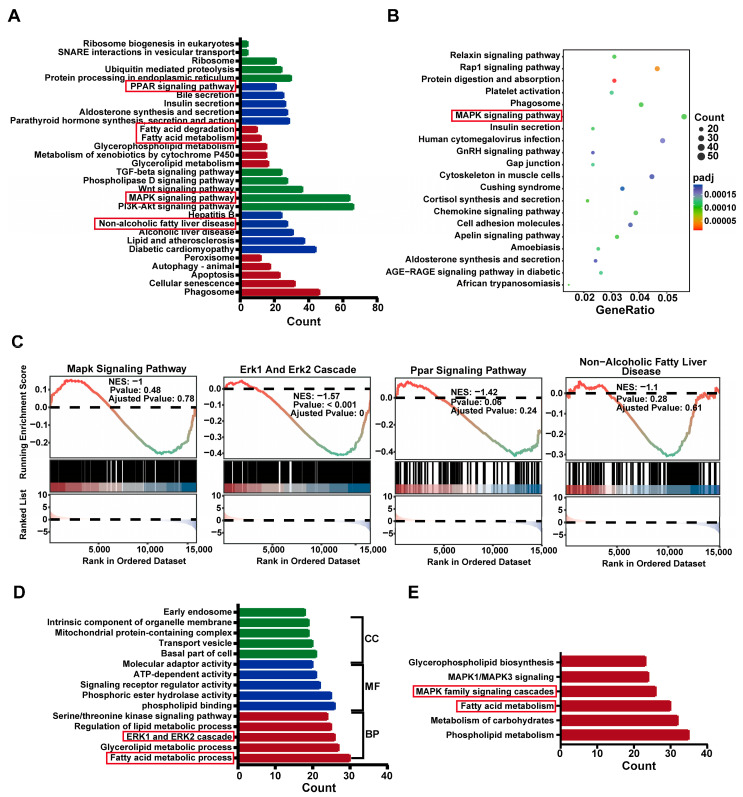
Enrichment of differentially expressed genes in the MAPK and PPAR signaling pathways in SHR liver: (**A**,**B**) KEGG functional analysis of differentially expressed genes; (**C**) GSEA analysis of the MAPK signaling pathway, ERK1 and ERK2 Cascade, PPAR signaling pathway, and non-alcoholic fatty liver disease; (**D**) GO functional analysis of differentially expressed genes; (**E**) Reactome functional analysis of differentially expressed genes.

When (P)RR is activated, the phosphorylation of its serine and tyrosine residues occurs, leading to the activation of downstream MAPK family members, such as ERK1/2 [19]. Activated ERK upregulates PPARγ, promoting lipid accumulation [20]. To further investigate the functional pathways associated with differential gene expression, we performed enrichment analysis on the differentially expressed genes from WKY and SHR liver tissues using Gene Ontology (GO), Kyoto Encyclopedia of Genes and Genomes (KEGG), and Reactome databases. KEGG analysis revealed that the differentially expressed genes were primarily enriched in MAPK, PPAR signaling pathways, and fatty acid metabolism (Figure 5A,B). GO analysis showed significant enrichment in the ERK1 and ERK2 cascade and fatty acid metabolic processes (Figure 5D). Reactome analysis further enriched these genes in MAPK family signaling cascades (Figure 5E). Gene Set Enrichment Analysis (GSEA) confirmed the important roles of the MAPK, ERK1, and ERK2 cascade and PPAR signaling pathways in lipid metabolism in SHR liver (Figure 5C). Collectively, these results suggest that the ERK cascade and PPAR pathways may be involved in hepatic lipid accumulation in SHRs.

### 2.6. Activation of the ERK/PPARγ Pathway in SHR Liver

**Figure 6:**
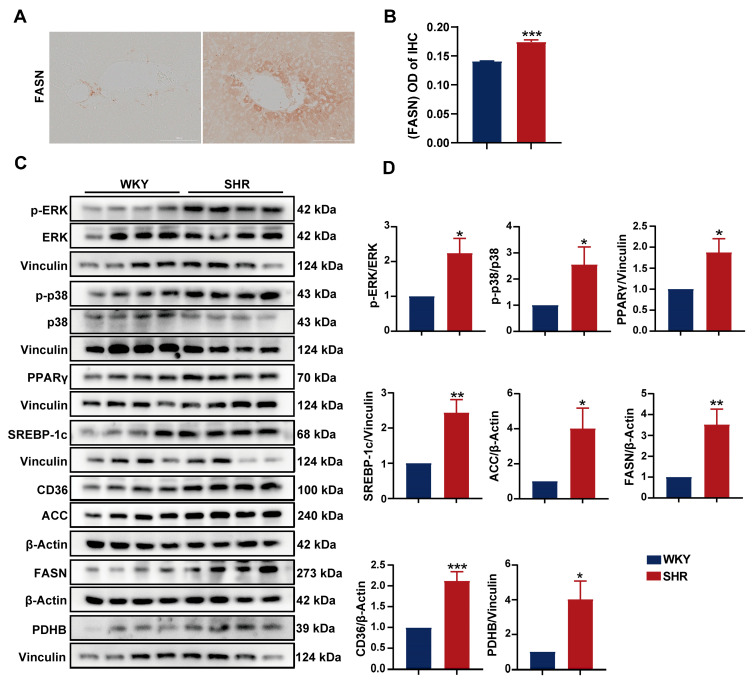
Activation of the ERK/PPARγ pathway in SHR liver: (**A**,**B**) IHC images and quantification of FASN expression (200× magnification); (**C**,**D**) protein expression of p-ERK, ERK, p-p38, p38, PPARγ, SREBP-1c, CD36, ACC, FASN, PDHX, and PDHB in SHR liver tissue. Data are presented as the mean ± SEM, analyzed by an unpaired two-sided *t*-test. * *p* < 0.05, ** *p* < 0.01, *** *p* < 0.001, compared with the WKY group.

To further investigate the impact of the ERK and PPAR pathways on lipid metabolism in SHR liver, we assessed the protein expression in SHR liver tissue. We observed a significant increase in the expression of (P)RR downstream proteins, including p-ERK, PPARγ, and p-p38. The elevated expression of PPARγ in the liver upregulates SREBP-1, which further activates the expression of several genes involved in fatty acid synthesis, such as acetyl-CoA carboxylase-1 (ACC-1) and fatty acid synthase (FASN) [26,27]. In SHR liver tissue, we found the significantly upregulated protein expression of SREBP-1, as well as its downstream fatty acid synthesis enzymes ACC-1, FASN, and the fatty acid transporter CD36 (Figure 6C,D), indicating increased fatty acid synthesis and transport in SHR liver. Pyruvate dehydrogenase (PDH), an enzyme responsible for converting pyruvate to acetyl-CoA, serves as a central metabolic node [28]. The PDH E1 component subunit β (PDHB) was significantly upregulated. The immunohistochemical analysis of SHR liver tissue revealed a significant increase in FASN expression (Figure 6A,B), consistent with the aforementioned results. These findings suggest the presence of the (P)RR/ERK/PPARγ axis in SHR liver, which promotes lipid accumulation.

### 2.7. Involvement of the (P)RR/ERK/PPARγ Pathway in Hypertension Combined with MAFLD

**Figure 7:**
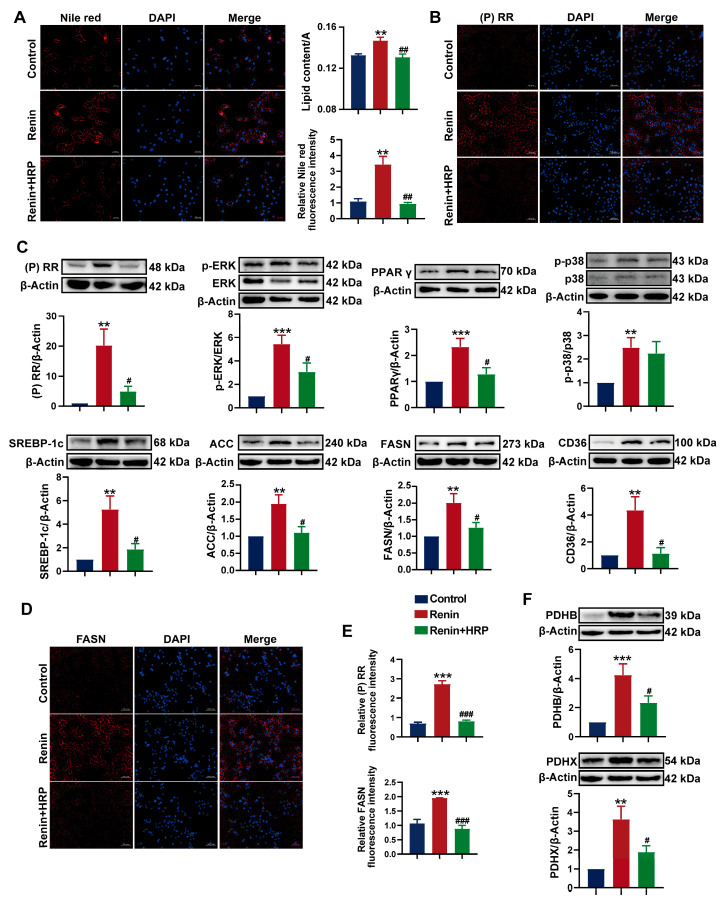
Specific inhibition of (P)RR reverses ERK/PPARγ pathway activation and reduces lipid accumulation: (**A**) Nile red (red) fluorescence staining to visualize lipid droplets in HepG2 cells (*n* = 3). DAPI (blue) stains the cell nuclei, with relative fluorescence intensity and lipid content quantification (*n* = 5). Images with 200× magnification; (**B**) IHC images of (P)RR expression (red), with DAPI (blue) staining the cell nuclei (*n* = 3). Images with 200× magnification; (**C**,**F**) effect of HRP on the protein expression of (P)RR, p-ERK, ERK, PPARγ, p-p38, p38, SREBP-1c, ACC-1, FASN, CD36, PDHB, and PDHX in HepG2 cells (*n* = 3); (**D**) IHC images of FASN expression (red), with DAPI (blue) staining the cell nuclei (*n* = 3). Images with 200× magnification; (**E**) relative fluorescence intensity of (P)RR and FASN immunofluorescence (*n* = 5). Data are presented as the mean ± SEM, analyzed by an unpaired two-sided *t*-test. ** *p* < 0.01, *** *p* < 0.001, compared with the control group. ^#^ *p* < 0.05, ^##^ *p* < 0.01, ^###^ *p* < 0.001, compared with the renin group.

**Figure 8:**
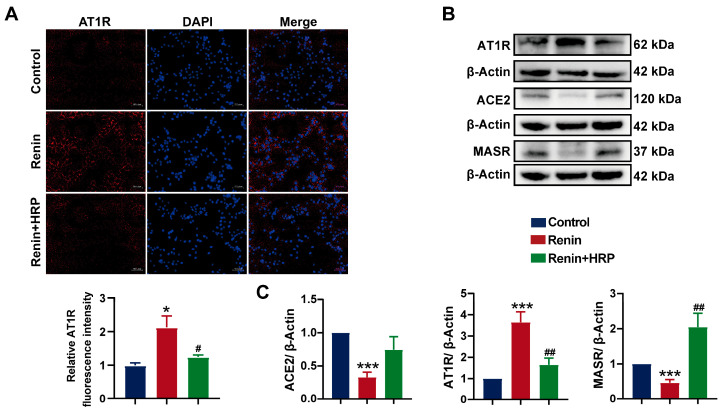
Specific inhibition of (P)RR reverses RAS activation: (**A**) immunofluorescence images of AT1R expression (red), with DAPI (blue) staining the cell nuclei, and relative fluorescence intensity (*n* = 5). Images with 200× magnification; (**B**,**C**) effect of HRP on the protein expression of MASR, AT1R, and ACE2 in HepG2 cells (*n* = 3). Data are presented as the mean ± SEM, analyzed by an unpaired two-sided *t*-test. * *p* < 0.05, *** *p* < 0.001, compared with the control group. ^#^ *p* < 0.05, ^##^ *p* < 0.01, compared with the renin group.

To further investigate the role of the (P)RR/ERK/PPARγ pathway in the development of hypertension combined with MAFLD, we used the (P)RR antagonist HRP for the specific inhibition of (P)RR. After the addition of HRP, Nile red fluorescence staining and Oil Red O staining quantification showed that HRP significantly reversed the increase in lipid content induced by renin in HepG2 cells (Figure 7A), confirming the key role of (P)RR in hypertension combined with MAFLD. Western blot analysis demonstrated that renin significantly upregulated the expression of (P)RR/ERK/PPARγ and their downstream proteins. However, HRP significantly inhibited the expression of (P)RR and downregulated the expression of its downstream proteins, including p-ERK and PPARγ. More importantly, HRP also significantly inhibited the expression of PPARγ downstream proteins, including SREBP-1c, ACC-1, FASN, and CD36 (Figure 7C,F), thereby reversing the lipid accumulation induced by renin in HepG2 cells. Immunofluorescence and quantitative analysis further confirmed these results (Figure 7B,D,E). Additionally, HRP significantly decreased AT1R protein expression and increased the expression of MASR and ACE2 (Figure 8B,C), reversing the renin-induced activation of RAS in HepG2 cells, which is closely related to the improvement of hypertension. The immunofluorescence results also showed that HRP decreased AT1R expression (Figure 8A). These findings suggest that the activation of the (P)RR/ERK/PPARγ pathway induces lipid accumulation in hepatocytes, further leading to the development of hypertension combined with MAFLD.

## 3. Discussion

As a common chronic disease worldwide, MAFLD affects more than one-third of the population. MAFLD often coexists with various systemic diseases [29]. The most prominent feature of MAFLD is systemic metabolic dysregulation. Both hypertension and MAFLD are key components of metabolic syndrome. Early-stage MAFLD is characterized by mild steatosis and lipid accumulation, which progressively worsen without intervention, accompanied by inflammation and fibrosis, eventually leading to hepatocellular carcinoma. Oxidative stress and mitochondrial dysfunction are both involved in the pathological process of MAFLD. The hepatic TG content is positively correlated with mitochondrial oxidative stress. In MAFLD patients, the activity of mitochondrial complexes I-V is reduced, and mitochondrial bioenergetic efficiency is impaired, which also promotes the generation of hepatic ROS, ultimately leading to mitochondrial dysfunction and progression of MAFLD. Therefore, inhibiting oxidative stress and maintaining mitochondrial function may become a therapeutic approach for this disease [30]. In MAFLD patients and animal models of different genders, the levels of saturated fatty acids in the liver are elevated. These can promote the synthesis of inflammatory factors and induce oxidative stress, which is a major cause of hepatocyte injury. Furthermore, with the progression of MAFLD, the levels of monounsaturated fatty acids also increase in liver tissue samples, while polyunsaturated fatty acids progressively decrease [31,32]. Hypertension is a chronic condition characterized by persistently elevated arterial blood pressure and involves complications in multiple organs, including the heart and kidneys [33]. Studies have shown a close association between hypertension and liver diseases. More than half of hypertensive patients are affected by MAFLD. One study reported that PPARα-deficient mice on a normal diet developed MAFLD, characterized by significantly increased hepatic triglyceride levels, while also exhibiting markedly higher arterial blood pressure compared to control mice [34]. Multiple studies have indicated that SHRs, a classic animal model for hypertension research, often exhibit marked hepatic lipid accumulation [23,35]. Regression analysis has revealed a significant positive correlation between hypertension and the incidence of MAFLD when systolic pressure ≥ 130 mmHg and diastolic pressure ≥ 80 mmHg [3]. The risk level of hypertension can be predicted through liver ultrasound, liver biopsy, and the assessment of liver injury biomarkers [36]. Hypertension and MAFLD often coexist and exhibit a complex bidirectional relationship, though the specific mechanisms linking them remain unclear [3].

(P)RR is widely expressed in various tissues and organs of the human body. The overactivation of (P)RR is associated with several diseases such as heart failure, diabetic nephropathy, albumin overload, and liver fibrosis. Studies show that the plasma s (P)RR levels in heart failure patients are significantly higher than in healthy individuals [37]. In the doxorubicin-induced heart failure model, (P)RR expression is increased [38]. This indicates that (P)RR is activated in heart failure diseases. Furthermore, the mechanism of cyclosporine A-induced nephropathy in rats is the (P)RR activation of RAS [39]. In db/db diabetic nephropathy (DKD) mice, (P)RR is highly expressed in the renal tubules, which shows a positive correlation with renal injury in clinical DKD patients. The specific knockout of renal (P)RR significantly improves inflammation and renal damage [40]. In pathological environments, renal (P)RR expression is often upregulated. In both humans and mice, (P)RR protein expression significantly increases in the context of liver fibrosis. The knockout of liver (P)RR genes reduces pro-fibrotic factors and inhibits the expression of fibrosis genes, delaying the progression of liver fibrosis [41]. (P)RR is activated to varying degrees in multiple organs, including the heart, kidneys, and liver, and in various diseases, indicating that (P)RR is closely related to the pathological development of diseases. Targeting (P)RR-based therapeutic strategies are expected to advance the diagnosis and treatment of various diseases.

(P)RR can bind and enhance renin activity, as well as fully activating the biologically inactive prorenin, thereby increasing the production of angiotensin II and inducing hypertension. Although the role of (P)RR in the induction and progression of hypertension has been extensively studied, its role and underlying mechanisms in MAFLD remain underreported. Studies have shown that a Fast Food Diet (FFD) induces hepatic steatosis in mice, with a significant increase in (P)RR expression. The specific inhibition of (P)RR can ameliorate hepatic steatosis [42]. (P)RR antagonists can inhibit the upregulation of hepatic PPARγ expression induced by a high-fat diet (HFD), improving hepatic steatosis and the progression of fibrosis. This confirms the involvement of (P)RR in hepatic triglyceride synthesis [43]. In this study, it was found that SHRs, while suffering from hypertension, also exhibited significant liver damage, as evidenced by markedly elevated serum ALT and AST levels, along with significantly increased hepatic TG and FFA levels. These findings are consistent with previously reported results. This indicates that the SHR model has hypertension combined with MAFLD. Since (P)RR is a classical member of the RAS system and is involved in the development of hypertension, we speculate that it may be involved in the lipid accumulation in SHR liver. (P)RR can participate in various signaling pathways, including MAPK, WNT/β-catenin, and phosphoinositide 3-kinase/AKT [44]. Upon (P)RR activation, serine and tyrosine residues are phosphorylated, further activating downstream ERK1/2 [45]. ERK phosphorylation activates PPARγ, promoting lipid accumulation [20,46]. Furthermore, (P)RR has also been identified as a direct target gene of PPARγ [47]. PPARγ is highly expressed in adipocytes, where it regulates lipid deposition. During the progression of MAFLD, higher concentrations of free fatty acids accumulate in the liver, including both saturated and unsaturated fatty acids, both of which can activate PPARγ [48]. High expression of hepatic PPARγ can increase the transcription of SREBP-1c, and simultaneously, PPARγ can directly promote the expression of FAS and CD36, facilitating TG synthesis and transport [26,49]. SREBP-1c is highly expressed in the liver, adipose tissue, and other tissues, where it activates multiple genes related to fatty acid synthesis, such as ACC and FAS [27]. We detected the expression of (P)RR proteins in SHR liver and found that it was significantly upregulated, promoting downstream ERK phosphorylation. (P)RR further upregulates the expression of PPAR-γ, SREBP-1c, and their downstream proteins, such as ACC, FAS, and CD36. This suggests the presence of the (P)RR/ERK/PPARγ axis in SHR liver to promote the occurrence and development of MAFLD.

The first (P)RR antagonist, HRP, is a bait peptide composed of 10 amino acids, corresponding to the sequence in the prorenin handle region [50]. HRP can specifically bind to (P)RR and inhibit its expression. A large number of studies have demonstrated the therapeutic potential of HRP in blocking (P)RR. (P)RR knockdown or HRP injection in FFD-induced MAFLD mice significantly inhibits (P)RR expression and reverses MAFLD and fibrosis [42]. After high-dose HRP treatment, the significant reversal of heart failure and lowered blood pressure were observed in chronic kidney disease mice, while also protecting the kidneys and alleviating kidney damage [51]. HRP blocking of (P)RR significantly reduced the myocardial infarction area in MI mice and alleviated fibrosis and hypertrophy [52]. Additionally, HRP injection can also treat preeclampsia (PE) in mice induced by reduced uterine perfusion pressure (RUPP) operation [53]. In summary, HRP has become a commonly used (P)RR antagonist.

**Figure 9:**
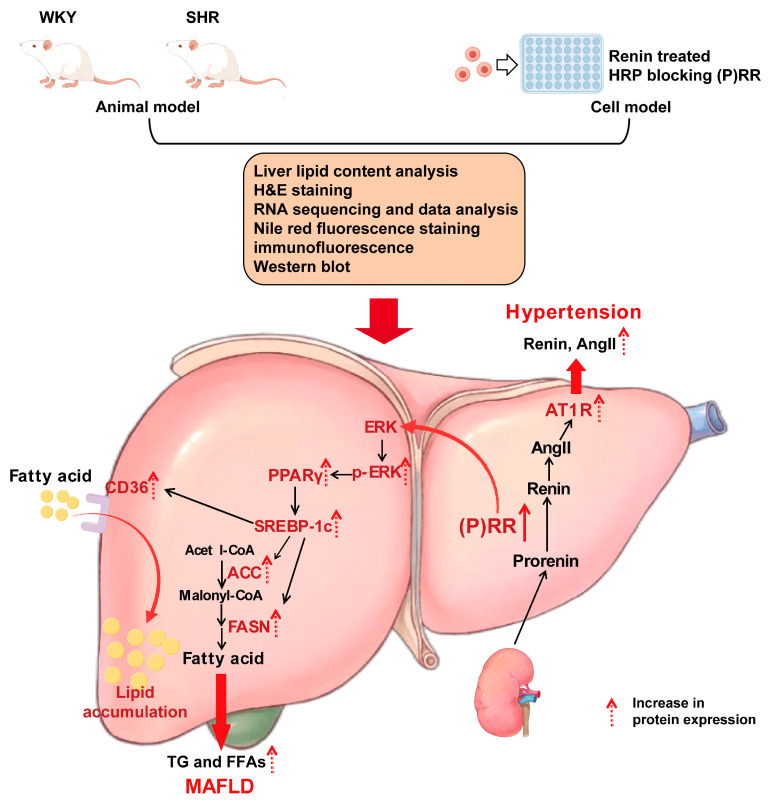
The (P)RR/ERK/PPARγ pathway plays an important role in the progression of spontaneous hypertension combined with MAFLD.

To clarify the intrinsic mechanisms of hypertension combined with MAFLD in SHRs and the relationship with the (P)RR/ERK/PPARγ pathway, we added HRP to specifically block (P)RR in in vitro experiments. We selected 10 nM human renin as the inducer [45] and established the renin-induced HepG2 cell lipid deposition model for the first time. Oil Red O staining and Nile red staining analysis both showed significant lipid deposition in HepG2 cells induced by renin. Western blot and immunofluorescence analysis showed that renin treatment significantly increased the expression of (P)RR, ACC-1, and AT1R proteins in HepG2 cells, while ACE2 and MASR were significantly downregulated. This suggests that the renin-induced lipid deposition model in HepG2 cells is similar to the local RAS activation accompanied by lipid accumulation in SHR liver. After adding HRP, AT1R protein expression decreased and MASR and ACE2 expressions increased, indicating that inhibiting (P)RR can reverse RAS activation. More importantly, HRP reversed the increase in lipid droplets in HepG2 cells induced by renin. Western blot analysis indicated that HRP could reverse the activation of the renin-induced (P)RR/ERK/PPARγ pathway. HRP significantly downregulated the expression of (P)RR, PPARγ, SREBP-1c, and their downstream proteins, such as ACC-1, FASN, and CD36, and inhibited ERK phosphorylation, but had no effect on p38. (P)RR is first revealed as a potential therapeutic target for hypertension combined with MAFLD. It has been confirmed that (P)RR can promote the expression of downstream proteins related to fatty acid synthesis and transport, such as ACC-1, FAS, and CD36, by upregulating the expression of ERK/PPARγ, thereby inducing the occurrence of hypertension combined with MAFLD (Figure 9).

Due to inherent species differences, the mechanism of hypertension combined with MAFLD based on the (P)RR/ERK/PPARγ axis, as observed in rat and cell models, may not fully translate to human pathophysiology. While animal and cell models provide valuable insights into the potential mechanisms of this disease process, they do not always replicate the complexity and variability of human physiology. Further studies in human-specific models or clinical trials are necessary to validate these findings.

## 4. Materials and Methods

### 4.1. Animals

Male spontaneous hypertension rats (SHRs) aged 6–8 weeks were used as the hypertension combined with MAFLD disease model, SHRs were used as the model group, and Wistar-Kyoto (WKY) rats were used as the control group. Both groups were given a standard diet without any intervention and were housed for 8 weeks. Euthanasia was performed by inhaling an overdose of anesthetics and exsanguination. An anesthesia machine with an induction chamber and waste gas removal system was used, with >4.5% isoflurane administered in oxygen until the animal stopped breathing for 60 s. The organs were then rapidly excised. All animals were provided by Beijing HFK Bio-Technology. Co., Ltd. (Beijing, China). The animals had free access to food and water and were housed in a ventilated room with a 12/12 h light/dark cycle. The temperature was maintained at 20–24 °C with 40–60% humidity. All experiments were conducted following the recommended guidelines for animal experiments and were approved by the Animal Ethics Committee of Peking Union Medical College, Chinese Academy of Medical Sciences (Approval No. 00004030, approval date 21 February 2024), in compliance with NIH guidelines for animal care and use.

### 4.2. Measurement of Blood Pressure

Blood pressure was measured using a tail cuff device (BP98AL, Softron, Tokyo, Japan) for WKY and SHR rats. The rats were restrained, and their tails were pre-warmed to 37 °C for 5–10 min, followed by the measurement of the tail artery pressure using an oscillometric cuff connected to the tail base. Systolic blood pressure was collected for each group of rats.

### 4.3. Histology Examination

Liver samples from each group of rats were fixed in 4% buffered paraformaldehyde. After treatment with ethanol and xylene, the samples were embedded in paraffin and sectioned. Hematoxylin and eosin (H&E) staining was performed, and the slides were observed under an optical microscope (3CCD, SONY, Tokyo, Japan).

Primary antibodies were incubated overnight on the slides at 4 °C. Secondary antibody incubation was performed for 2 h, followed by DAB staining. Pathological results were quantified using a microscope and ImageJ software (Image-Pro-Plus 7.0). Antibody information is shown in Supplementary Document Table S2.

### 4.4. Biochemical Analysis

We used an automated biochemical analyzer (TBA-40FR, TOSHIBA, Tokyo, Japan) to measure serum high density lipoprotein cholesterol (HDL-C), alanine aminotransferase (ALT), and aspartate aminotransferase (AST) levels in rats. Serum renin, angiotensin II (Ang II), and angiotensin 1–7 (Ang 1–7) levels were measured using an enzyme-linked immunosorbent assay (Jining Shiye, Shanghai, China). A reagent kit (Nanjing Jiancheng Technology Co., Ltd., Nanjing, China; Beijing Boxin Biotechnology Co., Ltd., Beijing, China) was used to measure the levels of total triglyceride (TG) and FFA in liver tissue homogenate supernatant.

### 4.5. RNA Sequencing and Data Analysis

Total RNA was isolated from the livers of WKY and SHR rats using Trizol reagent (Invitrogen, Carlsbad, CA, USA). The quantity and quality of the total RNA samples were assessed using a NanoDrop 2000 spectrophotometer (Thermo, Waltham, MA, USA). RNA with a 260/280 absorbance ratio of 1.8–2.2 was utilized for library construction and sequencing. Illumina HiSeq library construction was conducted according to the manufacturer’s instructions (Illumina, San Diego, CA, USA). mRNA sequencing was carried out using oligo dT primers to synthesize cDNA (APExBIO, Beijing, China). cDNA products were purified by magnetic beads. After library construction, the fragments were enriched through PCR amplification, selecting those in the size range of 350–550 bp. Library quality was evaluated using an Agilent 2100 Bioanalyzer (Agilent, Santa Clara, CA, USA). The library was sequenced using the Illumina NovaSeq 6000 sequencing platform. TrimGalore was employed to filter the raw paired-end fastq reads using the Cutadapt tool. Clean reads were aligned to the hg19 human genome using HISAT2, and transcriptome assembly and gene expression quantification were conducted with StringTie using reference genome-guided assembly. Differentially expressed genes (DEGs) were identified by DEseq2, where Log_2_ |multiple change| ≥ 0.25, *p* < 0.05 cut-off value. The functional enrichment analysis of significant DEGs and potential genes annotated in the identified modules was conducted based on GO and KEGG pathway classification using ClusterProfiler. Items with *p* values < 0.05 were considered significant. GSEA was conducted using the ClusterProfiler package 3.14.0 (Bioconductor, Boston, MA, USA).

### 4.6. Cell Viability Assay and Renin-Induced HepG2 Cells

Human hepatocellular carcinoma HepG2 cells were cultured in DMEM high-glucose medium containing 100 units/mL penicillin, 100 μg/mL streptomycin, and 10% heat-inactivated fetal bovine serum in a 37 °C incubator with 5% CO_2_.

HepG2 cells in the logarithmic growth phase were seeded into 96-well plates at a density of 8 × 10^4^ cells/mL, with 100 μL per well, and cultured for 24 h. The control group, renin model groups with concentrations of 10, 20, and 40 nM, and the HRP group were set. The HRP group was treated with 10 nM renin and 1 nM HRP, while the renin model and control group received an equal volume of DMSO. After 24 h of culture, cell viability was assessed using the MTT reagent. Following the same method, cells were fixed with cell fixative (Beijing Solabao Technology Co., Ltd., Beijing, China), stained with Oil Red O for 60 min, washed, and then 50 μL isopropanol was added to each well. After shaking to mix, the OD values were measured using a microplate reader (Mlutiskan FC, Thermo Fisher, Waltham, MA, USA).

### 4.7. Nile Red Fluorescent Staining

HepG2 cells in the logarithmic growth phase were seeded into 6-well plates at a density of 4 × 10^4^ cells/mL, fixed, and then stained with Nile red working solution (0.5 μg/mL) in the dark for 8 min. After discarding the dye, the cells were stained with DAPI for 5 min, followed by PBS washing. Fluorescence intensity in each group was observed under an inverted fluorescence microscope, and statistical analysis was performed.

### 4.8. Immunofluorescence and Nuclear Staining

Fixed cells were treated with 0.1% Triton X-100 for 15 min. The cells were incubated overnight with primary antibodies at 4 °C. FITC-conjugated secondary antibody was used to treat the cells for 1 h, and DAPI was used to stain the cell nuclei. Immunofluorescence images were captured using a fluorescence microscope. Antibody information is shown in Supplementary Document Table S2.

### 4.9. Western Blot (WB)

Liver tissue or HepG2 cell protein extracts were dissolved in RIPA buffer containing protease and phosphatase inhibitors (C1055, Applygen, Beijing, China). Protein quantification was performed using a BCA kit (P1511, Applygen, Beijing, China). Equal amounts of total protein were loaded onto SDS-PAGE gels. After electrophoresis, the proteins were transferred to Millipore (IPVH00010, Merck, Darmstadt, German) nitrocellulose membranes, which were then exposed to 5% milk for 2 h. The membranes were incubated overnight with primary antibodies. After washing three times with TBS/0.1% Tween-20, the membranes were incubated with specific secondary antibodies. ECL detection reagents (36208ES60, Yisheng Biotech Co., Ltd., Shanghai, China) were used, and the blot was scanned using a gel imaging system (OI-X6Touch, Guangzhou, China). Band densities were analyzed using Gel-Pro Analyzer 4.0 software (Media Cybernetics, Rockville, MD, USA). Antibody information is shown in Supplementary Document Table S2.

### 4.10. Statistical Analysis

All statistical analyses and graphs were generated using GraphPad Prism 8 for Windows (GraphPad Software Inc., San Diego, CA, USA). A *t*-test was used for statistical comparison between two groups, while one-way ANOVA followed by LSD post hoc test was used for multiple group comparisons. Data for protein expression levels of (P)RR, MAPK, PPARγ, FAS, SREBP-1c, ACC, MASR, AT1R, ACE2, PDHB, and CD36, as well as serum data with non-normal distribution (HDL-C, ALT, AST, TC, Ang II, Ang 1–7, TG, and FFAs), were analyzed. Data are presented as mean ± standard error, and a *p*-value of <0.05 was considered statistically significant. All experiments were repeated at least three times.

## 5. Conclusions

Our study first reveals that (P)RR is a potential therapeutic target for hypertension combined with MAFLD. And we found the (P)RR/ERK/PPARγ axis for the first time, which plays an important role in the progression of spontaneous hypertension combined with MAFLD. In vivo experiments show that (P)RR is highly expressed in the SHR liver and that the (P)RR/ERK/PPARγ axis is present in the liver. In vitro experiments confirmed that (P)RR plays a role between hypertension and MAFLD and that inhibiting (P)RR expression can improve hypertension combined with MAFLD via the ERK/PPARγ pathway. This study first reveals the molecular mechanism by which the (P)RR/ERK/PPARγ pathway participates in the development of hypertension combined with MAFLD, providing support for the development of future therapeutic drugs for hypertension combined with MAFLD.

## Data Availability

All results generated or analyzed during this study are included in this published article and Supplementary Materials. Data and materials will be made available upon request via email to the corresponding author.

## Supplementary Materials

The following supporting information can be downloaded at: https://www.mdpi.com/article/10.3390/ijms26136541/s1.

## Abbreviations

Abbreviation information is shown in Supplementary Document Table S1.
