# Supplementary material for: Overexpression of (P)RR in SHR and Renin-Induced HepG2 Cells Leads to Spontaneous Hypertension Combined with Metabolic Dysfunction-Associated Fatty Liver Disease
Source: Int J Mol Sci. 2025 Jul 7;26(13):6541. doi: 10.3390/ijms26136541 (PMC12250376; doi:10.3390/ijms26136541)
Supplement: Supplementary file 1 [file ijms-26-06541-s001.zip › ijms-3729307-supplementary.pdf]

Supplementary Information

Supplementary Table S1:Abbreviations

NAFLD	Non-alcoholic fatty liver disease
MAFLD	Metabolic dysfunction-associated fatty liver disease
RAS	Renin angiotensin system
(P)RR	(Pro) renin receptor
FFAs	Free fatty acids
MAPK	Mitogen activated protein kinase
PPAR-γ	Peroxisome proliferator-activated receptor gamma
FAS	Fatty acid synthase
SREBP-1c	Sterol regulatory element-binding protein 1c
ACC	Acetyl-CoA carboxylase
SHR	Spontaneous hypertension rats
WKY	Wistar-Kyoto
H&E	Hematoxylin and eosin
HDL-C	High density lipoprotein cholesterol
ALT	Alanine aminotransferase
AST	Aspartate aminotransferase
TC	Total cholesterol
Ang II	Angiotensin II
Ang 1-7	Angiotensin 1-7
TG	Total triglyceride
NAS	NAFLD activity score
SAF	Steatosis activity fibrosis
GO	Gene Ontology
KEGG	Kyoto Encyclopedia of Genes and Genomes
GSEA	Gene set enrichment analysis
MASR	Mas receptor
AT1R	Angiotensin II Type 1 receptor
ACE2	Angiotensin converting enzyme 2
PDHX	Pyruvate dehydrogenase complex component X
PDHB	Pyruvate dehydrogenase E1 component subunit beta
HRP	Handle Region Peptide
CD36	Cluster of Differentiation 36

Supplement Table S2. Reagents and antibodies

Reagent name	Cat.No	Origin
DMEM High Glucose	12100	Beijing Solarbio Science & Technology Co., Ltd.
Fetal bovine serum	16000-044	Gibco, USA

Tryspin-EDTA solution	T1300	Beijing Solarbio Science & Technology Co., Ltd.
(P)RR	A6531	ABclonal Technology Co.,Ltd., China
ACE2	A4612	ABclonal Technology Co.,Ltd., China
β-Actin	AC026	ABclonal Technology Co.,Ltd., China
AT1R	A4140	ABclonal Technology Co.,Ltd., China
MASR	A0230	ABclonal Technology Co.,Ltd., China
p-ERK	AP0485	ABclonal Technology Co.,Ltd., China
ERK	A4782	ABclonal Technology Co.,Ltd., China
p-p38	AP1311	ABclonal Technology Co.,Ltd., China
p38	A41771	ABclonal Technology Co.,Ltd., China
PPARγ	A19676	ABclonal Technology Co.,Ltd., China
SREBP-1c	A15586	ABclonal Technology Co.,Ltd., China
ACC-1	A15606	ABclonal Technology Co.,Ltd., China
FASN	3180S	Cell Signaling Technology, USA
CD36	A14714	ABclonal Technology Co.,Ltd., China
PDHB	A6943	ABclonal Technology Co.,Ltd., China
PDHX	A6426	ABclonal Technology Co.,Ltd., China
COL3A1	A3795	ABclonal Technology Co.,Ltd., China
Vinculin	A2752	ABclonal Technology Co.,Ltd., China
HRP Goat Anti Rabbit IgG(H+L)	AS074	ABclonal Technology Co.,Ltd., China
Anti-mouse IgG HRP-linked Antibody	7076	Cell Signaling Technology, USA
